# Four-Bar Linkage Path Generation Problems Using a New TLBO and Optimum Path Repairing Technique

**DOI:** 10.3390/biomimetics11030160

**Published:** 2026-02-28

**Authors:** Seksan Winyangkul, Mahmoud Alfouneh, Suwin Sleesongsom

**Affiliations:** 1 Department of Logistic Engineering and Management, Faculty of Industrial Technology, Chiang Rai Rajabhat University, Chiangrai 57100, Thailand; 2 Department of Mechanical Engineering, Zabol University, Zabol 98615-538, Iran; 3 Department of Aeronautical Engineering, International Academy of Aviation Industry, King Mongkut’s Institute of Technology Ladkrabang, Bangkok 10520, Thailand

**Keywords:** four-bar linkage, optimization, metaheuristics, TLBO, constraint handling technique

## Abstract

A self-adaptive variant of teaching–learning-based optimization, incorporating a diversity archive and referred to as ATLBO-DA, has been proposed. Combined with a new path repairing technique (PRT), it efficiently accomplishes the four-bar linkage path generation problem, but an upgraded version is needed. An update of ATLBO-DA to self-adaptive teaching–learning-based optimization with evenness factor archive (ATLBO-EFA) and a new path repairing technique are proposed at the present. The diversity archive idea of the original version is replaced with the evenness factor archive to increase the exploitation and exploration performance of the TLBO. An optimum path repairing technique (OPRT) is proposed. This novel approach is used to identify the optimum combination of four-bar mechanism types by employing the concept of Degree of Limiting (DL). Moreover, in this article, a comparative analysis of present update and the previous version use to solve four-bar linkage path generation problems is performed. Several path generation problems are solved using both techniques. The results demonstrate that the updated technique consistently outperforms the earlier version, giving superior values for both mean and minimum descriptive statistics. In addition, the results make it clear that ATLBO-EFA and OPRT are superior to the original version. The result of non-parametric statistic testing using Friedman test indicate that ATLBO-EFA ranks 1st at *p*-value (0.0455) < α (0.05). It can be concluded that ATLBO-EFA with OPRT offers the best solution for solving the four-bar path synthesis problems.

## 1. Introduction

The design of four-bar linkages (FBL) for path synthesis is crucial in various engineering applications including aircraft systems, mechanisms like landing gear, control surfaces, and flaps, which rely on complex linkages for smooth and reliable movement. Achieving optimal path synthesis for these systems requires advanced computational techniques to handle the intricate nonlinearities and constraints involved.

Four-bar linkage is a common mechanical mechanism that is applied in many devices used in daily life and production operations such as in front wheel suspensions [1], oil wells [2], and windshield wipers [3]. This mechanism’s original design may be divided into three categories. The most popular is ranking from path, motion, and function generation, respectively. The goal of an FBL path generation problem is to minimize the summed squared error (SSE) between predefined target points and the actual points traced by a coupler link. The problem is constructed as an optimization problem in which the mechanism’s link lengths and other parameters must be found.

To ensure the mechanism works as intended, certain conditions need to be met, like having a crank-rocker motion type, which requires specific relationships between the linkage lengths. These constraints make the optimization process more challenging because they limit the range of possible solutions and create a complicated search space. Traditional methods, like gradient-based approaches, struggle to handle these constraints and non-linearities effectively. Later, the optimization problem solving turned toward metaheuristic algorithms. Classical techniques such as Genetic Algorithms (GA), Differential Evolution (DE), and Teaching-Learning-Based Optimization (TLBO), etc., have been used in solving complex problems because they do not rely on gradients and easy to handle diverse constraints [4,5,6]. At present this group of techniques is called metaheuristics (MHs).

For constraint handling, the most popular approach for managing constraints in path synthesis problems in recent years is the penalty function (PF) method. To decrease the likelihood of selecting solutions that violate specific constraints in subsequent optimization iterations, this method imposes a large penalty by augmenting the objective function value of such solutions. But this method frequently results in an elimination of most of the initial population, if not all of them, particularly when strict guidelines, such as sequence constraints, are applied [7,8,9,10,11,12,13]. Because it usually takes a much larger population to find practical solutions, this problem can make optimization inefficient and considerably increase computational costs [14].

To address these challenges, researchers have devised multiple approaches to enhance sustainability and efficiency. In order to lessen the possibility of constraint violations in subsequent iterations, early work proposed methods to reorder input angles in the initial population [8]. Furthermore, a refinement procedure was proposed to reassign link lengths for individuals that do not satisfy Grashof’s criteria, which are essential for crank-rocker behavior. This was subsequently broadened by a different method that allows sequence conditions to be neglected until the very end of optimization by rearranging input angles both at initialization and at each iteration [12].

According to an additional study, the early optimization phases could be made simpler by creating an initial population that automatically satisfies Grashof’s conditions [13]. Whereas more recent study has developed ways to produce individuals that satisfy both Grashof’s law and sequence constraints, these approaches frequently produce completely new individuals instead of maintaining beneficial characteristics from earlier generations [10,11].

Despite their flexibility, metaheuristics still face issues with constraint handling in four-bar linkage problems. Common approaches, like penalty functions that add a cost to infeasible solutions, do not always work reliably, especially with linkage constraints that are highly specific. To address these limitations, recent studies have explored alternative methods, such as adjusting infeasible solutions to make them meet constraints, and a strategy known as path repairing technique (PRT) [4,5,6]. These techniques can increase computation time, but it guarantees a reliable result. Furthermore, more recent efforts have focused on adaptive optimization methods, where control parameters are adjusted dynamically during the search process to improve performance, which also approved increasing performance in design of the four-bar linkage path generation problems [4,5,6].

In this study, we propose a new approach for optimizing four-bar linkage path generation problem with a new optimum path repairing technique for handling constraint. Our method builds on the Teaching-Learning-Based Optimization (TLBO) algorithm, adding a self-adaptive mechanism and an evenness factor archive (ATLBO-EFA) in teaching phase, while a new optimum path repairing technique (OPRT) is improved with adding degree of limiting (DL). By dynamically adjusting parameters and maintaining a diverse set of solutions, we aim to improve both the reliability and quality of path synthesis results. This approach addresses some of the key challenges in the new optimizer and constraint handling and provides a better way to approach four-bar linkage design. Highlights of this research are:

(i) To propose a new self-adaptive variant of teaching–learning-based optimization, incorporating an evenness factor archive (ATLBO-EFA).
(ii) To propose a new optimum path repairing technique (OPRT).
(iii) A comparative study found that the new ATLBO-EFA with OPRT outperformed ATLBO-DA with PRT, which can enhance searching ability requiring for path generation problem in both precision and reliability.

## 2. Position Analysis of Four-Bar Linkages

**Figure 1:**
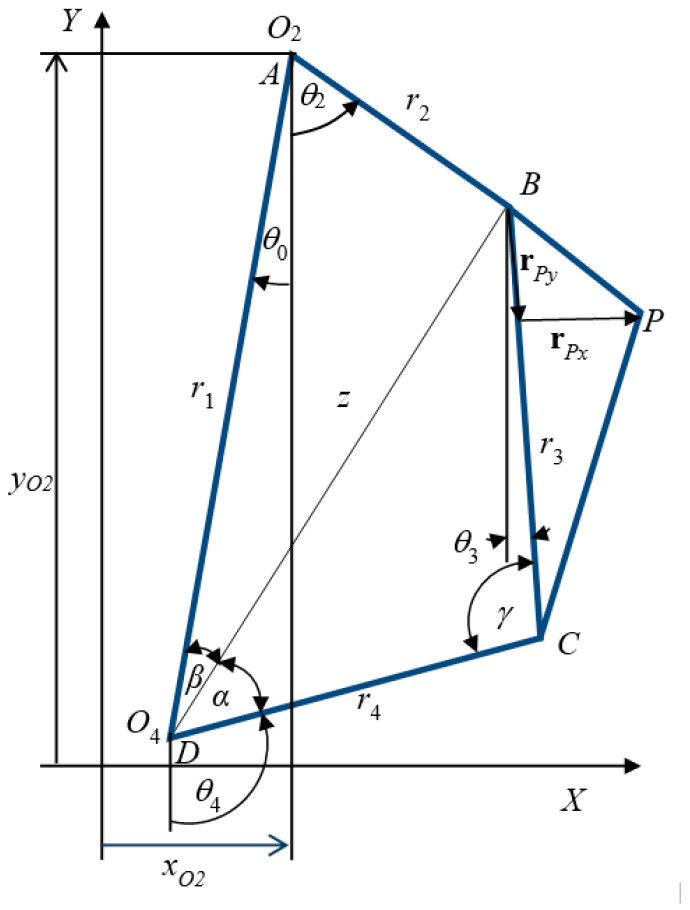
Kinematic diagram of a four-bar linkage in a global coordinate system.

A simple kinematic diagram for path generation problems of four-bar linkage is shown in Figure 1. It is composed of four simple links as its name suggests, which are connected with four revolute joints (name denoted by capital letters). One of the four links is designated as a frame and named as link number 1, and the remaining links are named, respectively, 2–4 as shown in Figure 1. Gruebler’s equation confirms this kind of linkage needs only a single input as it possesses one degree of freedom for a planar mechanism. This linkage is called a constrained chain mechanism. Its applications are related to its motion. The law used to classify its motion is called Grashof’s criterion. It is called type I, if the sum of shortest and longest links is less than the sum of remaining two links. Otherwise, it is called type II, if the former is greater than the latter. It is always a double rocker, which means there is no one linkage completely rotated. In cases where the sums are equal, it is a parallelogram.

In the case of the type I configuration, the mechanism is categorized as the crank-rocker. In this configuration, the shortest link serves as the input link where one of the links besides functions as a frame. Only input link can completely rotate a circle. It is a double rocker, if the link opposite to the shortest link is a frame. The last one is a double crank (both input and output links complete a circle, if the shortest link is set as a frame.

Linkage lengths (*r*_1_, *r*_2_, *r*_3_, *r*_4_) and other parameters are the four-bar linkage path generation problem design variables, which is called a dimensional synthesis design problem. The objective of this optimization problem is to minimize error of actual trace points (*x*_P_, *y*_P_) and desired path (*x*_d_, *y*_d_). The actual points (*x*_P_, *y*_P_) of traced point *P* are on the coupler link when the crank moves with complete cycle then the actual trace path is performed. For kinematic model, more details can be found in [14]. From Figure 1, a target point on coupler link in the global coordinate system is expressed as

(1) $$\begin{array}{l} x_{P}=x_{O2}+r_{2}\cos(\theta_{2}+\theta_{0})+L_{1}\cos(\varphi_{0}+\theta_{3}+\theta_{0}),\\ y_{P}=y_{O2}+r_{2}\sin(\theta_{2}+\theta_{0})+L_{1}\sin(\varphi_{0}+\theta_{3}+\theta_{0}) \end{array}$$

where *x_O_*_2_ and *y_O_*_2_ are the pin joint *O*_2_ coordinates in the global coordinate system. With using law of cosine to the couple link *BCP*, *ϕ*_0_ can be obtained:

(2) $$\varphi_{0}=\cos^{-1}\left[\frac{L_{1}^{2}+r_{3}^{2}-L_{2}^{2}}{2L_{1}r_{3}}\right]$$

From the above equations, objective function evaluation can be evaluated in a simple way. Distance z is measured in diagonal direction from point *O*_4_ to *B*, and it is used to calculate following variables. The values of angles *θ*_3_, *θ*_4_, *β*, *α* and γ depend on geometrical link lengths *r*_1_, *r*_2_, *r*_3_ and *r*_4_ at any crank angle (*θ*_2_), which is calculated as follows:

$$z^{2}=r_{1}^{2}+r_{2}^{2}-2r_{1}r_{2}\cos\theta_{2},\;z^{2}=r_{3}^{2}+r_{4}^{2}-2r_{3}r_{4}\cos\gamma ,$$

$$\gamma =\cos^{-1}\left[\frac{r_{3}^{2}+r_{4}^{2}-r_{1}^{2}-r_{2}^{2}+2r_{1}r_{2}\cos\theta_{2}}{2r_{3}r_{4}}\right],$$

$$\gamma =\cos^{-1}\left[\frac{r_{3}^{2}+r_{4}^{2}-z^{2}}{2r_{3}r_{4}}\right],\;\alpha =\cos^{-1}\left[\frac{z^{2}-r_{3}^{2}+r_{4}^{2}}{2zr_{4}}\right],$$

$$\beta =\cos^{-1}\left[\frac{z^{2}+r_{1}^{2}-r_{2}^{2}}{2zr_{1}}\right],\;\theta_{3}=\pi -(\alpha +\beta +\gamma ),$$

$$\theta_{4}=\pi -(\alpha +\beta ).$$

## 3. Optimization Problem and Constraint Handling

This research proposes a comparative test of a new variant of TLBO in solving path generation problems and a new PRT for managing constraint. The path generation problem can be expressed as an optimization task, and the objective here is to minimize the error between desired target curve and the actual trajectory traced by the coupler link, as shown in (3). The position error is derived in form of a sum squares error between each pair of points *P*_d_ and *P*_a_. *P*_d_ is the set of target or desired points, while *P*_a_ is the set of actual points. The design variables **x** is linkage geometry including *r*_1_, *r*_2_, *r*_3_, *r*_4_, target point position (*r_px_* and *r_py_*), the global position of *O*_2_ (*x_O_*_2_, *y_O_*_2_) and the angle of frame 1 (*θ*_0_). This kind of linkage can be controlled through input crank angle (*θ*_2_). The optimization problem that predefined input crank angles *θ^i^*_2_ is called a prescribed timing. If such input crank angles are included to design variables, the problem is called the without prescribed timing. In addition, the angle sequence constraints (ascending or descending order) are necessarily assigned in optimization problem to protect the mechanism from suddenly changing its direction motion (4). Furthermore, the second constraint is set to obtain linkage that corresponds to Grashof’s criterion (5)–(6). The remaining constraint is assigned for setting limit of design variables (7). The optimization problem can be expressed as

(3) $$f(x)=\sum_{i=1}^{N}\left[{\left(x_{d,i}-x_{P,i}\right)}^{2}+{\left(y_{d,i}-y_{P,i}\right)}^{2}\right]$$

Subject to:

*θ*_2_^1^ < *θ*_2_^2^ < … < θ_2_*^i^*< … < *θ*_2_*^N^* (4)

min(*r*_1_, *r*_2_, *r*_3_, *r*_4_) = crank (*r*_2_) (5)

2min(*r*_1_, *r*_2_, *r*_3_, *r*_4_) + 2max(*r*_1_, *r*_2_, *r*_3_, *r*_4_) < (*r*_1_ + *r*_2_ + *r*_3_ + *r*_4_) (6)

**x**_*l*_ ≤ **x** ≤ **x**_*u*_ (7)

where overall design variables vector is **x** = {x_O2_, y_O2_, r_1_, r_2_, r_3_, r_4_, r_px_, r_py_, *θ*_0_, *θ^i^*_2_}^T^, N is number of predefined points (*x*_d_, *y*_d_) on the prescribed or target curve and **x**_l_ and **x**_u_ are lower and upper limits of the design variables **x**, respectively.

## 4. Function Evaluation and Constraint Handling

**Figure 2:**
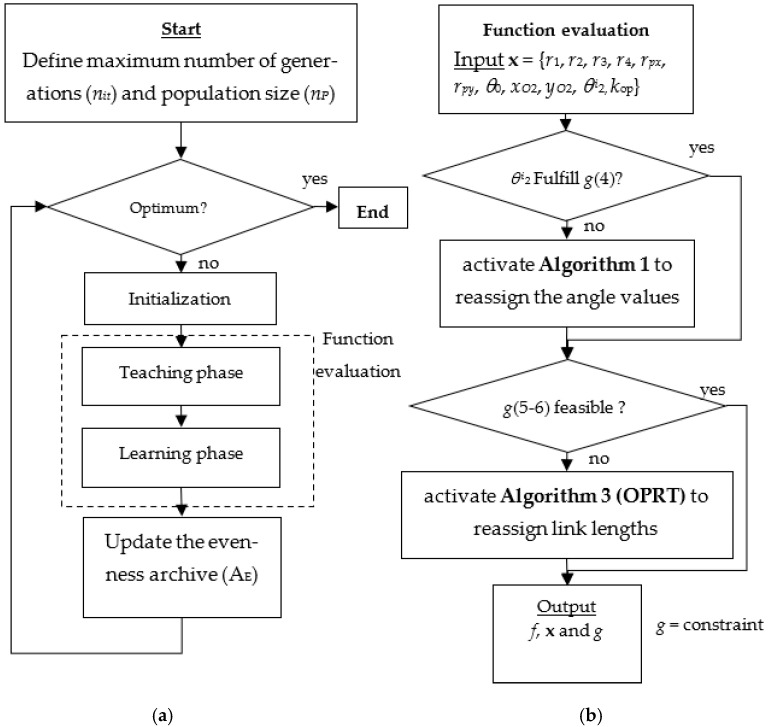
Flow diagram: (**a**) ATLBO-EFA; (**b**) function evaluation including OPRT.

How can we deal with constraints in solving with MHs? This was a question in the past. The traditional exterior penalty function was one option chosen in the past to handle the constraints. Unlike Deb’s feasibility rule, which introduces a more nuanced ranking of feasible and infeasible solutions, the exterior penalty method relies on a binary penalization mechanism. It functions like an on-off switch in an electric circuit [5]. If the constraints (4)–(6) do not fulfill at the solution x, each penalty parameter h_i_ of constraints (i = 1 … n) is added a large number to modify the objective function value. This state is an “on” state. Else, at an “off” state, h_i_ = 0. The objective is not modified with an additional penalty term. With such an idea, if h_i_ is assigned with a small value, the obtained solution is infeasible. Otherwise, if the number is too large, the optimizer will struggle to search for the optimum solution. These reasons make this technique inefficient and have motivated the previous work [4,5] to propose a new technique for constraint handling in path synthesis, but the previous technique still needs additional improvement. The previous technique is called the path repairing technique (PRT) [5], which has proved that its performance outperforms the traditional penalty technique. The PRT technique is composed of two parts. The first and second parts are concerned with repairing the link angle constraints (4) and repairing link length constraints (5)–(6), respectively. Previous workers [5] showed that introducing a combination of mechanism types within the population often yielded promising results. Populations containing a blend of crank–rockers, double–cranks, and double–rockers were shown to possess enhance diversity and solution quality. However, in general, this type of mixing cannot be built into the optimum design problem due to a lack of knowledge of the specific characteristics and function of each mechanism type. Thus, the contemporary technique still needs to be updated to make it more powerful in solving four-bar linkage path synthesis. The overall scheme of proposed techniques is presented in Figure 2, including constraint handling (Algorithm 1), function evaluation (Algorithm 2), OPRT (Algorithm 3), and ATLBO-EFA (Algorithm 4).

### 4.1. Input Link Angle Constraint

The path generation problem without prescribed timing requires an angle reassigning technique to protect the crank motion in a sudden change in direction. The algorithm is activated if its sequence obeys the constraint (4) as shown in Algorithm 1. The performance of this technique has been proven [5]. The reassigning technique will automatically update solution **x** to a new feasible design solution without constraint violation. First state of this technique, *N* uniform random numbers {*α*_1_, …, *α_N_*} are generated, where *α*_i_ ∈ (0, 1). If the sum of *α*_i_ > 2*π* radian, all values are scaled down so that the total does not exceed 2π. In this work, the sum is scaled down to less than one revolution at 1.99π. To generate an ascending input crank angle, the initial value of crank angle *θ*_2_^1^ is first assigned with *α*_1_ (unit in radian). Then, the next value of *α*_i_ for *i* = 2, …, *N* is accumulated to the next input crank angle until the last value is obtained as *θ*_2_^N^ = *θ*_2_^1^ + *α*_2_ + … + *α_N_*. With the reassigning technique, the new time sequence (all *N* input link angles) is absolutely rearranged in ascending order. Following this reordering, step, a portion of the design solution vector, corresponding to the input angles, is replaced with the updated set. For more information, please see [5].
**Algorithm 1.** Reassigning technique for timing constraint [5]Reassigning Technique for Timing ConstraintInput infeasible **x** at the elements $\theta_{2}^{i},\theta_{2}^{i+1},\;\;\ldots \;,\;\theta_{2}^{N}\;$ Output feasible **x** at the elements $\theta_{2}^{i},\theta_{2}^{i+1},\;\;\ldots \;,\;\theta_{2}^{N}\;$
Random numbers {*α*_1_,…, *α_N_*} are uniformly generated, *α_i_* ∈ (0, 1).If (*α*_2_ + … +*α_N_*) ≥ 2*π*, scale down all of them and modify each values as:                For *i* = 2 to *N*                      Generate *α_i_* = 1.99*πα_i_*/(*α*_2_ + … +*α_N_*)                EndFor *i* = 1 to *N*Step 1 If *i* = 1, *θ^i^*_2_ = *α*_1_.Step 2 Otherwise, *θ^i^*_2_ = *θ*_2_*^i^*^−1^ +*α_i_*.End

### 4.2. Grashof’s Criterion Constraint

A four-bar linkage can be categorized with Grashof’s criterion into two groups as Grashof’s and non-Grashof’s mechanism. In general, a crank rocker is in a group of Grashof’s mechanism, while the second group is a mechanism that fails to meet the crank rocker criteria. The second group is called non-Grashof’s mechanism. A crank rocker is commonly used in manufacturing applications, which requires only a single input crank that undergoes continuous full rotation. In such systems, the output link undergoes only partial or incomplete rotation. The geometric design of a four-bar mechanism must satisfy constraints derived from Grashof’s criterion in order to ensure its classification as either a double crank or crank rocker type. To check the possibility of usability, Grashof’s criterion can be transformed as per constraints (4)–(6).

If the geometry link length {*r*_1_, *r*_2_, *r*_3_, *r*_4_} violates the constraints (5)–(6), the new link length repairing technique is activated. This procedure proceeds according to Grashof’s criteria. A non-Grashof’s mechanism can be repaired to be a double-crank mechanism without requiring a penalty function by applying the repairing technique, which reassigns the link lengths in accordance with Grashof’s criterion. This technique can increase the likelihood of discovering improved solutions during optimization search, unlike the traditional penalty approach, which simply eliminates non-Grashof mechanism from consideration. Firstly, the numbers {*δ*_1_, *δ*_2_, *δ*_3_}, *δ_i_* ∈ (0,1) are generated uniformly at random, which presents the auxiliary variables as provide the basis for subsequent calculations, as outlined below.

*S*_1_ = *δ*_3_ (8)

*S*_2_ = *δ*_1_ (9)

*S*_3_ = *δ*_1_ + *δ*_2_ (10)

*S*_4_ = *δ*_1_ + *δ*_2_ + *δ*_3._ (11)

The new auxiliary variable (AV) *S_i_* is used in analysis instead of *r_i_* due to its proportional relationship.

According to the above Equations (8)–(11), if *δ*_3_ < *δ*_1_, the shortest link is *S*_1_ and the longest link is *S*_4_. Thus, the constraint (6) is holding if

2*δ*_3_ < *δ*_1_ (12)

This condition guarantees that the design mechanism functions as a double crank (moderately desirable case) by assigning the shortest link (*S*_1_) as frame. Otherwise (worst-case), if condition (12) is not satisfied, and the crank-rocker and double-crank never occurred, and the mechanism fails to achieve continuous rotation. To avoid the worst-case scenario in which a double rocker configuration is continually obtained, the AVs are modified when the condition 2*δ*_3_ > *δ*_1_ is met, as shown as follows:

*S*_1_ = *δ*_1_ (13)

*S*_2_ = 2*δ*_3_ (14)

*S*_3_ = 2*δ*_3_ + *δ*_2_ (15)

*S*_4_ = 2*δ*_3_ + *δ*_2_ + *δ*_1_ (16)

If conditions (13)–(16) are satisfied, the mechanism may operate as a double-rocker. In this case, condition (6) still holds if *δ*_1_ < *δ*_3_. Basically, MHs search for an optimum through iterative reproduction based on population evolution. Maintaining diversity within the populations often leads to improved results. In addition, in the real world, an effective population often consists in several crank rockers (most desirable), some double-cranks (second most desirable), and a few double-rockers (least desirable). The performance of such systems has been proved in [5]; however, further in-detail research is still needed. To avoid the attainment of a double rocker configuration, when the condition *δ*_3_ > *δ*_1_, is met, the AVs can modify as shown as follows:

*S*_1_ = *δ*_1_ (17)

*S*_2_ = *δ*_3_ (18)

*S*_3_ = *δ*_3_ + *δ*_2_ (19)

*S*_4_ = *δ*_3_ + *δ*_2_ + *δ*_1_ (20)

According to the above Equations (17)–(20), if *δ*_3_ is longer than *δ*_1_, the shortest and longest links are *S*_1_ and *S*_4_, respectively. Thus, the condition (6) holds if

2*δ*_1_ < *δ*_3_ (21)

The above Equation (21) introduces an additional condition that restricts the occurrence of non-Grashof’s mechanism. In parallel equations (14) and (18) quantify the extent of this restriction, expressed in the form of the degree of limiting (DL), denoted as *k*_op_ as shown as follows.

*S*_2_ = *k*_op_*δ*_3_ (22)

where *k*_op_ = (1,2). The degree of limiting (DL) factor is utilized to regulate the inclusion of non-Grashof’s mechanism within the population, enhancing the exploratory capability of the metaheuristic algorithm as previously discussed. The key question is whether or not the DL factor provides the most effective solution to the problem. Answering this requires an optimization design approach, which systematically evaluates its impact on both search performance and solution quality. Then the optimization problem in Equations (3)–(7) can be rearranged to include the *k*_op_ variable in a simple way. It is included as one of the original design variables. The function evaluation (3)–(7) as an algorithm is also shown in Algorithm 2.
**Algorithm 2.** Function evaluationFunction EvaluationInput **x** = { *r*_1_, *r*_2_, *r*_3_, *r*_4_, *r_px_*, *r_py_*, *θ*_0_, *x_O_*_2_, *y_O_*_2_, *θ^i^*_2,_ *k*_op_} Output *f*, **x** and constraints ***Evaluate constraints*** Step 1. In case optimization problem is the without-prescribed-timing problem and if *θ^i^*_2_ cannot fulfill constraint (4), activate Algorithm 1 to reassign the angle values. Step 2. If constraints (5–6) are infeasible, activate Algorithm 3 to reassign link lengths. ***Position analysis and function evaluation*** Step 1. Otherwise, solve Equations (2)–(7) for all values of *θ*_2_ and solve Equation (1) for **r**_p_ at each *θ*_2_. Step 2. Compute the objective function values and constraints according to Equations (4)–(7).

Algorithm 3 will be detailed in next.

The auxiliary variables including DL factor are modified as follows:

*S*_1_ = *δ*_1_ (23)

*S*_2_ = *k*_op_*δ*_3_ (24)

*S*_3_ = *S*_2_ + *δ*_2_ (25)

*S*_4_ = *S*_3_ + *δ*_1_ (26)

If we hold the conditions (23)–(26), it means that there is a possibility of having a mixture variety of four-bar mechanism types in population. Meanwhile, the optimum DL factor for each problem is determined in the process of solving the FBL path generation task. However, the solving optimum DL factor for repairing mechanisms can introduce nested problems, which may lead to unsuccessful runs. At this point, the successful run of OPRT led to the implementation of the present upgrade of the ATLBO-EFA. This task will be detailed in the results and discussions. Moreover, the optimum factor DL will be discussed in the design results section. With the optimum path repairing technique, Algorithm 3 is used to allow the necessary double rockers to be included in the population evolution with optimum DL factor.
**Algorithm 3.** Repairing Grashof’s criterion constraint with the optimum path repairing techniqueOptimum Repairing the Grashof’s Criterion ConstraintInput infeasible {*r*_1_, *r*_2_, *r*_3,_ *r*_4,_ *k_op_*}Output feasible {*r*_1_, *r*_2_, *r*_3,_ *r*_4,_ *k_op_*}Step 1. A set of numbers {*δ*_1_, *δ*_2_, *δ*_3_}, *δ_i_* ∈ (0,1) is generated uniformly at random. Step 2. If the constraints (5–6) are violated.Step 3. Assign values. *S*_1_ = *δ*_1_ *S*_2_ = *k_op_δ*_3_ *S*_3_ = *S*_2_ + *δ*_2_ *S*_4_
*= S*_3_ + *δ*_1_ Step 4. If max(*S*_1_, *S*_2_, *S*_3_, *S*_4_) > 1, compute *δ_i_* = *δ_i_*/2 and compute step (3) until max(*S*_1_, *S*_2_, *S*_3_, *S*_4_) < 1 Step 4. Compute *r_i_* = *r_min_* + (*r_max_* − *r_min_*)*S_i_* for *i* = 1, …, 4.

The last step in Algorithm 3 converses the value *S_i_* to *r_i_*.

Next, we look at an example of the use of Algorithm 3. Here, Algorithm 3 is applied by randomly generating set of four-bar linkage dimensions {r1, r2, r3, r4} = (5, 60) five trials. The generated four-bar linkage dimensions metric is as follows:

$${\left[r_{1}\;r_{2\;}\;r_{3}\;r_{4}\right]}^{T}=\left[\begin{array}{ccccc} 57.1681 & 55.7495 & 20.9226 & 51.6144 & 36.3427\\ 47.3164 & 35.2147 & 22.5519 & 30.2991 & 15.2488\\ 18.8837 & 40.9425 & 54.7345 & 24.4822 & 39.3775\\ 8.2695 & 43.9372 & 17.7668 & 43.1215 & 30.3183 \end{array}\right]$$

Checking Grashof’s criterion (6) for all dimensions reveals that the sets in columns 1 and 5 satisfy the condition, thereby passing the Grashof’s criterion check. The percentage of Grashof’s mechanism is 40% and non-Grashof’s mechanism is 60%. In more detail, the four-bar mechanism corresponding to columns 1 and 5 are classified as crank rocker type due to *r*_1_ (frame) of both dimensions is longer than *r*_2_ (crank). However, it is still possible to obtain a double crank type if the random *r*_1_ (frame) is shorter than *r*_2_ (crank). In general, the probability of generating a crank rocker and double crank is approximately 0.5. For the remaining dimensions that did not satisfy Grashof’s criterion, Algorithm 3 is applied to regenerate possible dimensions. The process begins by considering the probability of transforming a non-Grashof mechanism into a Grashof mechanism, as expressed in the conditions (23–26) below:

S_1_ = δ_1_

S_2_ = k_op_δ_3_

S_3_ = k_op_δ_3_ + δ_2_

S_4_ = k_op_δ_3_ + δ_2_ + δ_1_

It is possible to satisfy Grashof’s criterion (6) if S1 + S4 < S2 + S3 holds, the mechanism type is a double crank. The requirement for this improvement can be stated as:

2δ_1_ < k_op_δ_3_

If *k_op_* = 1, the condition is met with (21):

2δ_1_ < δ_3_

The probability of transforming a non-Grashof’s mechanism into a Grashof’s mechanism is 0.25 (remaining probability of the non-Grashof’s mechanism is 0.75).

If kop = 2, the condition is

δ_1_ < δ_3_

The probability of generating a Grashof’s mechanism is 0.5 (a half of non-Grashof’s mechanism).

In conclusion, the probability of generating a Grashof’s mechanism is (0.25,0.5) if *k_op_* = (1,2). The processing in OPRT can be utilized to search for the optimum degree of limiting (DL) factor, which serves to limit the occurrence of non-Grashof mechanisms. After performing OPRT, we see in the present example the generation of a mixture of mechanism types: crank–rocker mechanisms account for approximately 40%, double–crank mechanisms range between 15–30%, and double–rocker mechanisms range between 30–45%. The proportions of double–crank and double–rocker mechanisms can be adjusted through the application of the optimum DL factor. Thus, the proportions of double–crank and double–rocker mechanisms can be adjusted through the application of the optimum DL factor.

Follow step 1, the parameters {*δ*_1_, *δ*_2_, *δ*_3_}, *δ_i_* ∈ (0,1) are randomly generated for 3 times, as follows:

$${\left[\delta_{1}\;\delta_{2\;}\;\delta_{3}\;\right]}^{T}=\left[\begin{array}{ccc} 0.7135 & 0.5370 & 0.2563\\ 0.8439 & 0.1462 & 0.8956\\ 0.7530 & 0.4067 & 0.1177 \end{array}\right]$$

In Step 3, the auxiliary variables are calculated by considering three distinct cases that depend on the degree of limiting factor *k*_op_ = {1, 2, 1.5}. This division facilitates the study of the characteristics of the limiting behavior under different levels of restriction.

For example, *δ_i_* in column 1 and *k*_op_ = 1 are chosen to perform step 3:

S_1_ = δ_1_ = 0.2563

S_2_ = k_op_δ_3_ = (1)(0.7530) = 0.7530

S_3_ = S_2_ + δ_2_ = 0.7530 + 0.8439 = 1.5969

S_4_ = S_3_ + δ_1_ = 1.5969 + 0.2563 = 1.8532

Check step 4, if max(*S*_1_, *S*_2_, *S*_3_, *S*_4_) > 1, compute *δ_i_* = *δ_i_*/2 and repeatedly perform step 3.

δ_i_ = 0.1281, 0.4219, 0.3765 where i = 1, …, 3.

S_1_ = δ_1_ = 0.1281

S_2_ = k_op_δ_3_ = (1)(0.2034) = 0.3765

S_3_ = S_2_ + δ_2_ = 0.3765 + 0.4219 = 0.7984

S_4_ = S_3_ + δ_1_ = 0.7984 + 0.1281 = 0.9265

Check step 4, if max(*S*_1_, *S*_2_, *S*_3_, *S*_4_) < 1, Stop.

The repairing technique in Algorithm 3 is used to limit the mechanism type, which is fulfilling the Grashof criterion (S1 + S4 < S2 + S3). The double rocker can change to a double crank if *S*_1_ < *S*_2_.

Next is a continuation of the example but with *δ_i_* in column 2 and *k*_op_ = 2 selected to perform step 3:

S_1_ = δ_1_ = 0.4067

S_2_ = k_op_δ_3_ = (2)(0.5370) = 1.0740

S_3_ = S_2_ + δ_2_ = 1.0740 + 0.1462 = 1.2202

S_4_ = S_3_ + δ_1_ = 1.2202 + 0.4067 = 1.6269

Check step 4, if max(*S*_1_, *S*_2_, *S*_3_, *S*_4_) > 1, compute *δ_i_* = *δ_i_*/ 2 and perform step 3

δ_i_ = 0.2034, 0.0731, 0.2685 where i = 1, …, 3.

S_1_ = δ_1_ = 0.2034

S_2_ = k_op_δ_3_ = (2)(0.2685) = 0.5370

S_3_ = S_2_+ δ_2_ = 0.5370 + 0.0731 = 0.6101

S_4_ = S_3_+ δ_1_ = 0.6101 + 0.2034 = 0.8135

Check step 4, if max(*S*_1_, *S*_2_, *S*_3_, *S*_4_) < 1, Stop.

In Algorithm 3, the repairing technique is applied to restrict the mechanism type classified as a double-rocker to be a double crank (*S*_1_ < *S*_2_), which is fulfilled with the Grashof’s criterion (S1 + S4 < S2 + S3).

In the last case, the values *δ_i_* in column 3 and *k*_op_ = 1.5 are chosen for operation with OPRT. Under these conditions, the mechanism cannot be regenerated to satisfy the Grashof criterion since the inequality 2*δ*_1_ < *k_op_δ*_3_ is not satisfied. Then, it is a double rocker configuration (non-Grashof’s criterion) characterized by *S*_1_ < *S*_2_. Moreover, the value of the double rocker can be changed to that of double crank (*S*_1_ < *S*_2_) using the evolutionary technique, giving the second most desirable type for the path generation problem.

Then, the mixing of the crank rocker, double crank and double rocker (*S*_1_ < *S*_2_) can occur by switching the degree of limiting (DL) using the OPRT algorithm.

## 5. A New TLBO

### 5.1. TLBO

The performance of teaching-learning-based optimization (TLBO), which was proposed by [15] in 2011 as a tool for solving a wide range of engineering problems was judged favorably in previous research [4,5,14,16,17,18,19,20,21,22,23,24,25]. The performance of TLBO in solving path generation problems has been proved as has its ability to outperform others [4,5,14]. The TLBO performance is attributed to being parameter-less, which is one of the main features of this algorithm that makes it superior to some other algorithms. This technique is a population-based algorithm that mimics the teaching and learning processes that occur in general classroom teaching. It has two main reproduction processes, which start with the teacher phase, which is followed by the learning phase. In the first phase, students study and receive knowledge from a teacher; however, they cannot acquire all they need to know. In the learning phase, the students learn from their group study, and interaction with their peers, and their knowledge base builds accordingly. By analogy, in the case of the algorithm, iterations of the search process are reproduced through both phases, and the best solutions identified are then selected to form the next generation. The teacher’s phase starts with a class with a group of *n_P_* students, which denotes a design solution.

### 5.2. Self-Adaptive TLBO with an Evenness Factor Archive (ATLBO-EFA)

The present technique is improved from self-adaptive TLBO with diversity factor archive (ATLBO-DA) [5]. The original TLBO, teaching and learning phases are employed to create two new populations of equal sizes by two different reproduction operators. Both phases have different mission, the teaching phase emphasizes the search intensification, while the learning phase emphasizes the diversification. Premature convergence may occur at the teaching phase if the teaching operator relies on the position of the best solution. In this work, the probability of premature convergence similar to that reported in [5]—can be reduced by employing a group of teachers. The teachers are maintained within an evenness factor archive rather than a diversity factor archive. This archive stores a set of teachers designed to balance exploitation and exploration Such balance is achieved by mixing individuals from both the current and previous populations. Next, Pareto solutions (non-dominated solutions) of the bi-objective optimization problem are iteratively stored in the archive, and the two objectives dealt with are the original objective function (*f*(x)) and the evenness factor objective function (*f_EV_*). The evenness objective function values for 2*N_P_* solutions are calculated as follows:

(27) $$f_{E}(x)=w_{1}f(x)+w_{2}f_{2}(x)$$

where *w*_i_, *i* = 1, … 2, are weighting factors and *w*_1_ + *w*_2_ =1.

**Figure 3:**
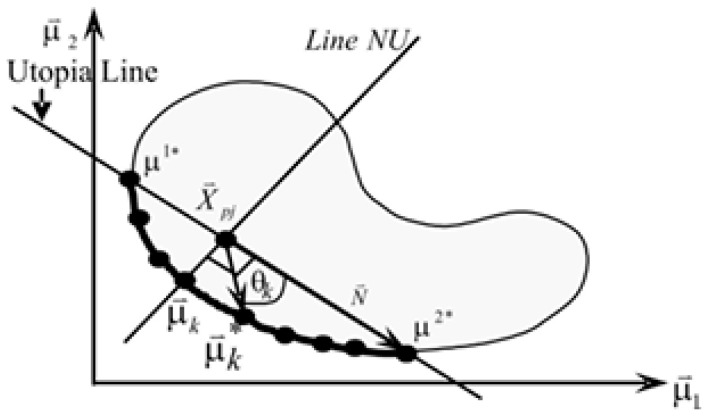
Distribution evenness factor.

The second objective function *f*_2_ of the union set of solution x*^i^* in the previous and current populations can be computed based on the following previous work [26], as can be seen in Figure 3:

(28) $$EV_{k}=\cos\theta_{k}=\frac{\vec{N}\cdot ({\vec{\mu }}_{k}-{\vec{X}}_{pj})}{\left|\vec{N}\right|\left|\left({\vec{\mu }}_{k}-{\vec{X}}_{pj}\right)\right|}$$

**Algorithm 4.** ATLBO-EFA algorithm**ATLBO-EFA Algorithm**Input: Define maximum number of generations and population size in form of *n_it_* and *n_P_*, respectively. Output: x^best^, *f*^best^ Initialization: Step I. *n_p_* initial students {x*^i^*} are generated and function evaluations {*f^i^*}. Step II. Generate four matrices: *LRR_Success*, *LRR_Fail TRR_Success*, and *TRR_Fail*, size 1 × 2, which is initially start with each element is unity. Main procedure Step 1. While termination criterion is not met, do the inner loop. {*Teacher Phase*} Step 2. Calculate M*_avg_* (the mean position of solutions {x*^i^*}). Step 3. Calculate *T_RR_* (the probabilities of choosing intervals) $PTRR_{j}=\frac{TRR\_Success_{j}}{TRR\_Success_{j}+TRR\_Fail_{j}}$. Step 4. For *i* = 1 to *n_P._* Step 4.1. Perform roulette wheel selection with *PTRR_j_*. Step 4.1.1. If *j* = 1 is selected, *T_RR_* = 0.4 + 0.1 *rand* is sampled. Step 4.1.2. Else, if *j* = 2 is selected, *T_RR_* = 0.5 + 0.1 *rand* is sampled. Step 4.2. Generate *P_r_* = *rand* and select a teacher. Step 4.2.1. If *P_r_* ≤ *T_RR_*, set the best solution as a teacher M_best_. Step 4.2.2. Else, if *P_r_* > *T_RR_*, randomly select a solution in A*_E_* and set it as a teacher M_best_. Step 4.3. Create x*^i^*_new_ using following formular and perform the evenness objective function evaluation (27). x_*new*_ = x_*old*_ + *Difference*_*Mean* where *Difference*_*Mean_i_* = *r_i_* (M*_i_*_, best_ − *T_f_*M*_i_*_,avg_), and *T_f_* = round(1 + *r*_i_). Step 4.3.1. If x*^i^*_new_ is better than x*^i^*, add 1 point to the *j*-th element of *TRR_Success*. Step 4.3.2. Else, add 1 point to the *j*-th element of *TRR_Fail*. Step 5. Replace {x*^i^*} by *n_P_* best solutions from {x*^i^*}∪{x*^i^*_new_}. {*Learning Phase*} Step 6. Calculate *L_RR_* (the probabilities of selecting intervals), which is similar to *T_RR_* in step 3. Step 7. For *i* = 1 to *n_P_* Step 7.1. Perform roulette wheel selection with *PLRR_j_*. Step 7.1.1. If *j* = 1 is selected, *L_RR_* = 0.4 + 0.1 *rand* is sampled. Step 7.1.2. Else, if *j* = 2 is selected, *T_RR_* = 0.5 + 0.1 *rand* is sampled. Step 7.2. Generate *P_r_* = *rand*. Step 7.2.1. If *P_r_* ≤ *L_RR_*, create x*^i^*_new_ using two-student learning and perform function evaluation. Step 7.2.2. Else, create x*^i^*_new_ using three-student learning and perform function evaluation. Step 7.3. Update *LRR_Success* and *LRR_Fail*. Step 7.3.1. If x*^i^*_new_ is better than x*^i^*, add 1 point to the *j*-th element of *LRR_Success*. Step 7.3.2. Else, add 1 point to the *j*-th element of *LRR_Fail*. Step 8. Replace {x*^i^*} by *n_P_* best solutions from {x*^i^*}∪{x*^i^*_new_}. Calculate the evenness objective function value of x*^i^*_new_(27). Step 9. Update the evenness archive (A_E_) with the non-dominated solutions obtained from {x*^i^*}_old_∪{x*^i^*}_new_. Step 10. End While.where *μ*_1_ = *d_ij_*, it is needed to calculate the maximum Euclidean distance between x*_i_* and x*_j_*,

(29) $$|d_{\mathrm{ij}}|=\sqrt{{\left(x_{i}\right)}^{2}+{\left(x_{j}\right)}^{2}}\;\;i,j\;=\;1,\ldots ,\;n(n-1)/2$$

*μ*_2_ = *f*(*x*) is the original objective function. The new design criteria (2-d) can be used to calculate the evenness factor. The distribution evenness factor lies between 0 and 1 such that the factor closes to 0 means that the solution set is more even, but the factor closes to 1 means that the solution set is less even.

The ascending order ranking is needed, then the minimum is presented as the evenness factor:

(30) $$f_{2}\;=\;EF=\min(EV_{k})$$

With such an indicator, the less crowded and high evenness is represented by the lower *f*_2_, thus it is a good selection for exploration. Meanwhile, the search direction is guided by *f*_1_. By combining these objectives, the algorithm simultaneously preserves direction and exploration. This balance is achieved through the weighted sum technique in (27). Therefore, the weighted sum in the function *f_E_* is a balance between exploitation and exploration. However, the values of *w*_1_ and *w*_2_ are randomly generated to create diversity for *f_E_*. The weighted sum has been studied with sensitivity analysis and it has been shown that it plays a crucial role in the computation of the ranking of alternatives, which makes this technique able to enhance the diversity of the population [27,28]. A new merit function vector is defined by f(x) = {*f*(x), *f_E_*(x)}*^T^*, which follows the non-dominated sorting process.

To judge whether x_1_ dominates x_2,_ if at least one element of f(x_1_) is strictly lower than the corresponding element in f(x_2_) (f(x_1_) ≤ f(x_2_) for all elements). ATLBO-EFA uses similar scheme for choosing teachers in the self-adaptive TLBO in the way non-dominated solutions are properly set as teachers.

Then the teacher can be either the best solution or randomly chosen one from the evenness factor archive. At this state, the teaching reproduction rate *T_RR_* is used to determine the probability of choosing the best solution as a teacher. On the other hand, based on the work by [5], with respect to the learning phase, the learning between two students is replaced by self-learning by three students. The self-learning by three students is achieved in such a way that three solutions are randomly selected from the current population. The best of them is assigned as x*^p^* while the other two are randomly assigned as x*^i^*^1^ and x*^i^*^2^ as shown in (31).

x*^i^_new_* = x*^i^_old_* + *rand*(*rand*(x*^p^* − x*^i^*^1^) + *rand*(x*^p^* − x*^i2^*)) (31)

where a uniform random number is generated by *rand* ∈ [0, 1]. This relationship is used to maintain population diversity in the three-student operator.

## 6. Numerical Experiments

Four path synthesis test problems of four-bar are used to evaluate the performance of the proposed technique and the previous improvement. Performance testing of the new approach is compared with the base-line technique (ATLBO-DA) and the previous results [4,5,14,29]:

**Problem** **1.** *Path generation without prescribed timing (straight line target path)* 

*Design variables:* 

**x** = {*r*_1_, *r*_2_, *r*_3_, *r*_4_, *r*_*px*_, *r*_*py*_, *θ*_0_, *x*_2_, *y*_2_,*θ*_2_^1^,*θ*_2_^2^,*θ*_2_^3^, *θ*_2_^4^, *θ*_2_^5^, *θ*_2_^6^, *k*_op_}^*T*^.

*Target points are r_d_^i^ = {(20,20), (20,25), (20,30), (20,35), (20,40), (20,45)}* 

*Limits of the design variables:* 

5 ≤ *r*_1_, *r*_2_, *r*_3_, *r*_4_ ≤ 60

−60 ≤ *r_px_*, *r_py_*, *x*_2_, *y*_2_ ≤ 60

0 ≤ *θ*_0_, *θ*_2_^1^,*θ*_2_^2^,*θ*_2_^3^, *θ*_2_^4^, *θ*_2_^5^, *θ*_2_^6^ ≤ 2*π*.

**Problem** **2.** *Path generation problem with prescribed timing (circular target path)*

*Design variables:*

**x** = {*r*_1_, *r*_2_, *r*_3_, *r*_4_, *r_px_*, *r_py_*, *θ*_0_, *x*_2_, *y*_2_, *k*_op_}^*T*^

$$\theta_{2}^{i}=\left[\frac{\pi }{6},\frac{\pi }{3},\frac{\pi }{2},\frac{2\pi }{3},\frac{5\pi }{6},\pi \right]$$

*Target points are r_d_^i^ = {(0,0), (1.9098,5.8779), (6.9098, 9.5106), (13.09, 9.5106), (18.09,5.8779), (20,0)}.*

*Limits of the design variables are:*

5 ≤ *r*_1_, *r*_2_, *r*_3_, *r*_4_ ≤ 50

−50 ≤ *r_px_*, *r_py_*, *x*_2_, *y*_2_ ≤ 50

$$0\;\leq \;\theta_{0}\;\leq \;2\pi .$$

**Problem** **3.** *Path generation problem without prescribed timing (elliptical target path)*

*Design variables are*

**x** = {*r*_1_, *r*_2_, *r*_3_, *r*_4_, *r_px_*, *r_py_*, *θ*_0_, *x*_2_, *y*_2_,*θ*_2_^1^,*θ*_2_^2^,*θ*_2_^3^, *θ*_2_^4^, *θ*_2_^5^, *θ*_2_^6^, *θ*_2_^7^, *θ*_2_^8^, *θ*_2_^9^, *θ*_2_^10^, *k*_op_}^*T*^.

*Target points are:*

*r_d_^i^ = {(20,10), (17.66,15.142), (11.736,17.878), (5,16.928), (0.60307,12.736), (0.60307,7.2638), (5,3.0718), (11.736, 2.1215), (17.66,4.8577), (20,10)}.*

*Limits of the design variables are:*

5 ≤ *r*_1_, *r*_2_, *r*_3_, *r*_4_ ≤ 80

−80 ≤ *r_px_*, *r_py_*, *x*_2_, *y*_2_ ≤ 80

0 ≤ *θ*_0_, *θ*_2_^1^,*θ*_2_^2^,*θ*_2_^3^, *θ*_2_^4^, *θ*_2_^5^, *θ*_2_^6^, *θ*_2_^7^, *θ*_2_^8^, *θ*_2_^9^, *θ*_2_^10^ ≤ 2*π*

**Problem** **4.** *A GA–DE Hybrid Evolutionary Algorithm for Path Synthesis of Four-Bar Linkage.*

*Design variables are*

**x** = {*r*_1_, *r*_2_, *r*_3_, *r*_4_, *r_px_*, *r_py_*, *θ*_0_, *x*_2_, *y*_2_,*θ*_2_^1^, *k*_op_}^*T*^.

*Target points are:*

r_d_^i^ = {(0.5,1.1), (0.4,1.1), (0.3,1.1), (0.2,1.0), (0.1,0.9), (0.005,0.75), (0.02,0.6), (0.0, 0.5), (0.0,0.4), (0.03,0.3), (0.1,0.25), (0.15,0.2), (0.2,0.3), (0.3,0.4), (0.4,0.5), (0.5,0.7), (0.6,0.9), (0.6,1.0)} and θ_2_^i^ = [θ_2_^1^, θ_2_^1^ + 20^°^*i] (i = 1,2, …, 17)

*Limits of the design variables are:*

0 ≤ *r*_1_, *r*_2_, *r*_3_, *r*_4_ ≤ 50

−50 ≤ *r_px_*, *r_py_*, *x*_2_, *y*_2_ ≤ 50

0 ≤ *θ*_0_, *θ*_2_^i^ ≤ 2*π*

The optimizers used to tackle the test problems are the original ATLBO-DA [5] and newly developed meta-heuristics (ATLBO-EFA) with optimum DL. The optimization input parameter is set as population size *n_P_* = 100, while the total number of iterations or generations for each optimization run is set to be 500. Its termination criterion is set up with the total number of function evaluations as 100 × 500. All problems are run 30 times to measure their convergence rate and consistency of both algorithms.

## 7. Design Results

The optimum dimensions of the four-bar linkage are the main purpose of the path generation of four-bar mechanisms, which minimize the objective function. Four case studies with and without prescribed timing are considered for performance testing of the new optimizer and optimum path repairing technique. The minimum value of the objective represents its performance in searching for finding the best optimum point, while the mean values are used as the reliable indicator, the higher the mean, the less reliable optimizer. The optimum DL factor is represented the best mixing of mechanism type for each problem case.

**Figure 4:**
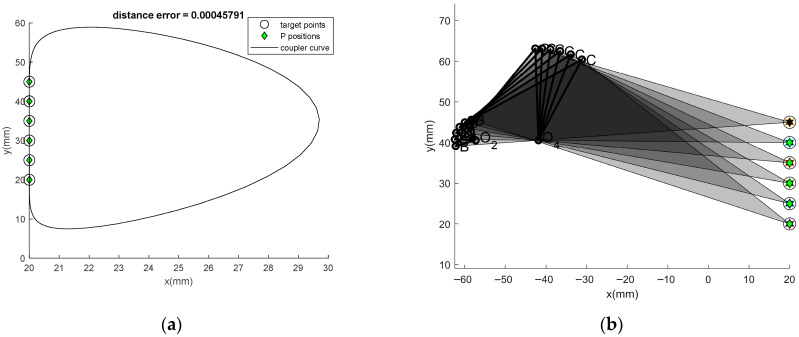
(**a**) Best coupler curve obtained in Problem 1. (**b**) Best mechanism obtained in Problem 1.

The results of Problem 1, which are obtained using the comparative optimizers together with the new optimum path repairing technique, are presented in the first three columns of Table 1. In the table, 30 optimization runs are used for descriptive statistics including: Mean (the mean objective function values), Max (the worse result), Min (the best result), and Std (the standard deviation). The table also indicates the best design variables identified by each algorithm. The statistic test is computed from the sum of squares of the distances between the desired points, and the actual points, while the term “Error” is the mean sum of squares of the distances between them. The error can be compared with the previous report. From the results, it is seen that the proposed method and the path repairing technique is far superior to those of the previous technique. From all 30 runs of Problem 1, the optimizer that gives the best results is ATLBO-EFA for both the mean value (*f_obj_* = 0.092709) and the best min (*f_obj_* = 1.5 × 10^−6^). The new technique can strongly reduce Max and Std. For Problem-1, the optimum DL factor converges to value of 1.7929 for ATLBO-DA and 1.0449 for ATLBO-EFA. This means that the OPRT transforms a double rocker into double crank with probabilities of occurrence close to 0.5 and 0.25, respectively. The higher value corresponds to poorer performance in this case, meaning that the closer the DL factor is to the lower bound, the better the outcome. Higher performance is achieved by regenerating with the addition of Grashof’s mechanism (double crank) for 26.12% of the non-Grashof mechanism type. Ultimately, the optimum degree of limiting (DL) aligns with the final mechanism choice for this case, identified as the crank rocker (Figure 4). Although link 2 can rotate in a complete a circle, it works partially in a circle. This indicates that the optimum mechanism operates as a double rocker, according to the selected optimum degree of limiting (DL). The chosen DL maintains the mixing of the double rocker as close as possible to its highest feasible limit. Significantly, the new technique is able to enhance consistency in convergence toward the optimum solution. Thus the new technique gives more reliable results than did the previous. The optimum path traced by the coupler point of the best solution and the kinematic diagram of the best linkage is shown in Figure 4a and Figure 4b, respectively. It is found that ATLBO-EFA with OPRT gives the best result (Error = 4.58 × 10^−4^) compared to the original version (0.0026) in the present run, which are better than the results reported in the literature [5,11,12], including the constraint handling with Deb’s feasibility rule [29]. The best four-bar linkage obtained from the best solution as shown in Figure 4b can completely rotate in an ascending direction.

**Figure 5:**
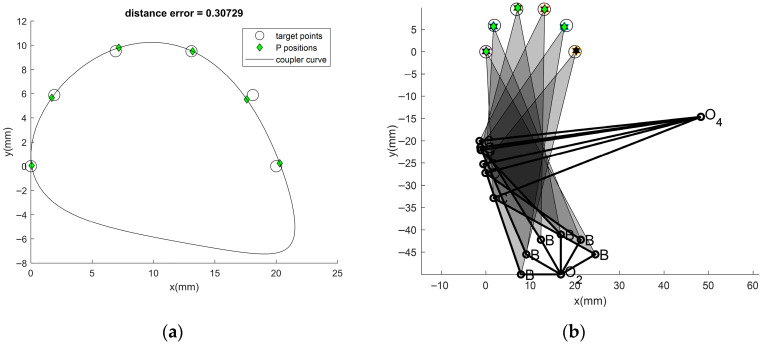
(**a**) Best coupler curve obtained in Problem 2. (**b**) Best mechanism obtained in Problem 2.

For Problem 2 with the number of target points at 6, the results obtained from using the previous ATLBO-DA and the new technique with OPRT are shown in Table 1, columns 4–6. The coupler curves and the best linkages obtained from TLBO are shown in Figure 5a and Figure 5b respectively. In this case, the new approach demonstrates a significant improvement in the mean and min, as well as the other statistic parameters, compared to previous results. For this case, the optimum DL converge to 1.0609 for ATLBO-DA and 1.2082 for ATLBO-EFA. Both approaches demonstrate a similar convergence trend, though with slightly different DL values. For this problem, a slightly elevated value of DL achieves better results. In this case, the value of *k*_op_ converges close to the lowest bound, which is reflected in the optimum DL trend. This means that the OPRT algorithm transforms the double rocker into a double crank with a probability close to 0.25. By regenerating with the addition of Grashof’s mechanism (double crank) for 30.20% of the non-Grashof mechanism type. The optimum DL aligns with the final optimum mechanism choice for this case, confirming that the best solution is crank rocker. Although link 2 can rotate in a complete a circle, it operates in a partial circle (Figure 5b). This means that the optimum mechanism functions as a double rocker, determined by the selected optimum degree of limiting (DL). The chosen DL keeps the mixing of the double rocker slightly below its highest allowable limit. As was the case in Problem-1, the new technique can increase consistency in convergence to the optimum solution better than the previous technique can [5,11,12]. From all 30 runs of Problem 2, the optimizer that gives the best mean is ATLBO-EFA (*f_obj_* = 6.814966), while the best result (*f_obj_* = 0.761388) is obtained from the present technique. The new technique can strongly reduce Max and Std rather than the previous variant [present run, 5, 11,12]. For Problem-2, each optimizer yields the best error value of 0.3073, demonstrating improved consistency across runs. The four-bar linkage obtained from the best solution, illustrated in Figure 5b, is capable of completing a full rotation in the ascending direction.

**Figure 6:**
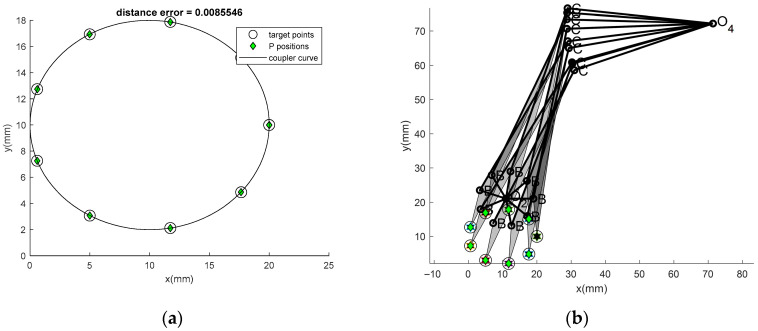
(**a**) Best coupler curve obtained in Problem 3. (**b**) Best mechanism obtained in Problem 3.

In Problem 3 with the prescribed curve with 10 points, the results obtained by the new algorithms with the optimum path repairing technique are shown in Table 2 (first three columns of the table). The coupler curve and the best linkage are shown in Figure 6a and Figure 6b, respectively. From the results, it is found that ATLBO-EFA gives the best results for both the mean objective function value and the best result when using OPRT. For this case, the optimum DL factor converges to 1.0149 for ATLBO-DA and 1.9105 for ATLBO-EFA. Unlike the earlier cases, here the higher value corresponds to better performance, indicating that ATLBO-EFA achieves superior results when the DL factor trends toward the upper limit. In this case, *k*_op_ converges close to the highest value, which is reflected in the optimum DL trend. This means that the OPRT algorithm transforms the double rocker into a double crank with probabilities close to 0.25 and 0.5, respectively. Higher performance is achieved by regenerating with the addition of Grashof’s mechanism (double crank) for 47.76% of the non-Grashof mechanism type. The optimum DL therefore aligns with the final optimum mechanism of choice for this case, which is crank rocker. In this case, link 2 is able to rotate through a complete circle, achieving full rotation (Figure 6b). This shows that the optimum mechanism functions as a true crank-rocker, determined by the selected optimum DL. The chosen DL keeps the mixing of the double rocker close to the lower limit, ensuring that the mechanism remains stable. Then here, the new techniques is better able to increase consistency in converge to the optimum solution than the previous technique [5,11,12]. From all 30 runs of Problem 3, the optimizer that gives the best mean is ATLBO-EFA (*f_obj_* = 0.291322), while the best result (*f_obj_* = 9.54 × 10^−4^) is obtained from the present technique. Similarly to the previous problems, the new technique greatly improved its performance in searching. The best result from ATLBO-EFA achieves an error of 0.0086, which is clearly superior to that of the original version (0.0234) in the present run. In addition, this performance is also superior to previous results reported in the literature [5,11,12], including the constraint handling with Deb’s feasibility rule [29]. The linkage obtained from the best solution as shown in Figure 6b can completely rotate in an ascending direction.

**Figure 7:**
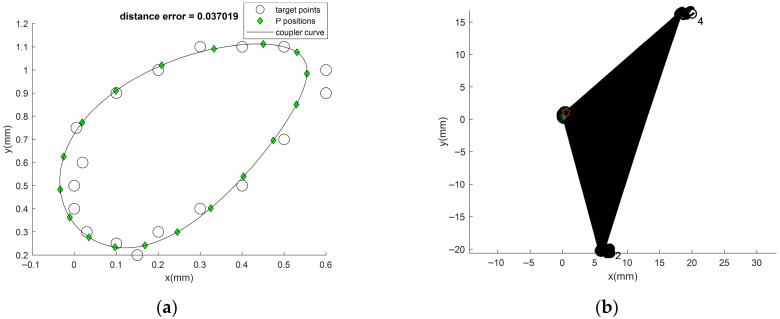
(**a**) Best coupler curve obtained in Problem 4. (**b**) Best mechanism obtained in Problem 4.

In Problem 4 with the prescribed closed-loop curve, the results obtained by the new algorithms with the optimum part repairing technique are shown in Table 2 (fourth–sixth column of the table). The coupler curve and the best linkage are shown in Figure 7a and Figure 7b, respectively. From the results, it is found that ATLBO-EFA gives the best results for both the mean objective function value and the best result when using OPRT. The optimum DL converges to value for ATLBO-DA (*k*_op_ = 1.8124) and ATLBO-EFA (*k*_op_ = 1.1989) in such a way that the lower becomes better. This case, the OPRT algorithm transforms the double rocker into a double crank with probabilities close to 0.5 and 0.25, respectively. Superior performance is thus achieved by transformation with the addition of Grashof’s mechanism (double crank) for 29.97% of the non-Grashof type. Although link 2 is capable of completing a full rotation, in practice it operates only partially within a circle (Figure 7b). This means that the optimum mechanism operates as a double rocker, determined by the selected optimum degree of limiting (DL). The chosen DL keeps the mixing of the double rocker slightly below its highest limit, in a manner similar to Problem 2. Furthermore, it means that the new technique can better increase consistency of convergence to the optimum solution than the previous technique [5,11,12]. From all 30 runs of Problem 2, the optimizer that gives the best mean is ATLBO-EFA (*f_obj_* = 0.286989), while the best result (*f_obj_* = 0.029717) is obtained from the present technique. Similarly to the previous problems, the new technique greatly improves its performance in searching. The best result from ATLBO-EFA gives the best error of 0.037, which is better than original version (0.039) in the present run and better than the previous result in the literature [5,11,12]. The linkage obtained from the best solution as shown in Figure 7b can completely rotate in an ascending direction in accordance with the elliptic target path.

The previous tables rely on descriptive statistic discussions, the non-parametric statistic test with the Friedman test is presented in Table 3. It is shown that ATLBO-EFA with OPRT is superior to the ATLBO-DA OPRT. In this study, the Friedman test and the Tukey–Kramer test are used for comparing the results. Based on the Friedman test, ATLBO-EFA ranks 1st at *p*-value (0.0455) < α (0.05), as shown in Table 3. It can be summarized that ATLBO-EFA is the best performer for solving the four-bar path synthesis problems cases 1–4. Based on the Tukey–Kramer test, the mean column ranks of both ATLBOs are significantly different.

Computational time required by the new method is approximated twice as the previous version as shown in Table 4, but the performance of the present is strongly increased as shown in Table 1 and Table 2. The new technique offers high returns, but it requires high investment. Then, the computational time is less significant than the performance.

**Figure 8:**
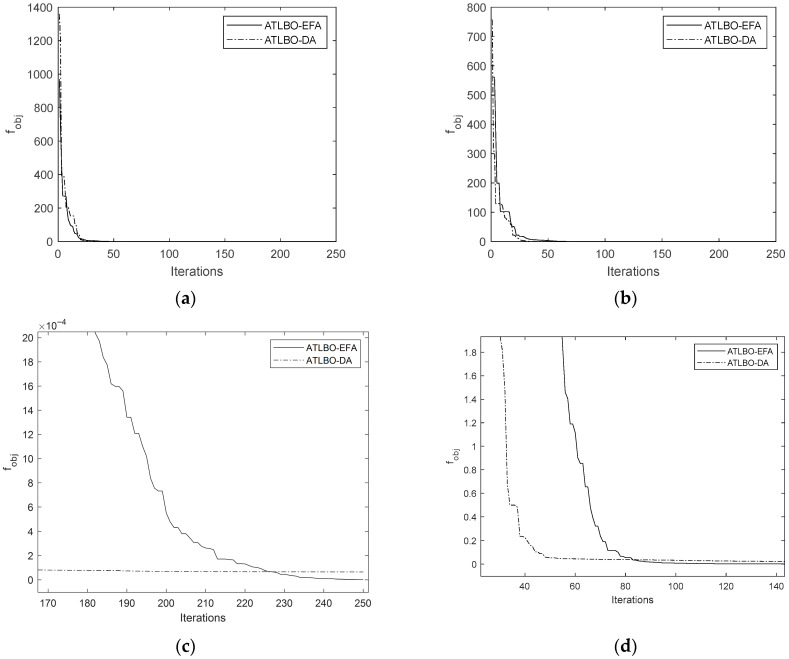
Search history of ATLBO-DA and ATLBO-EFA. (**a**) Problem 1. (**b**) Problem 2. (**c**) Enlarged search history of Problem 1. (**d**) Enlarged search history of Problem 2.

**Figure 9:**
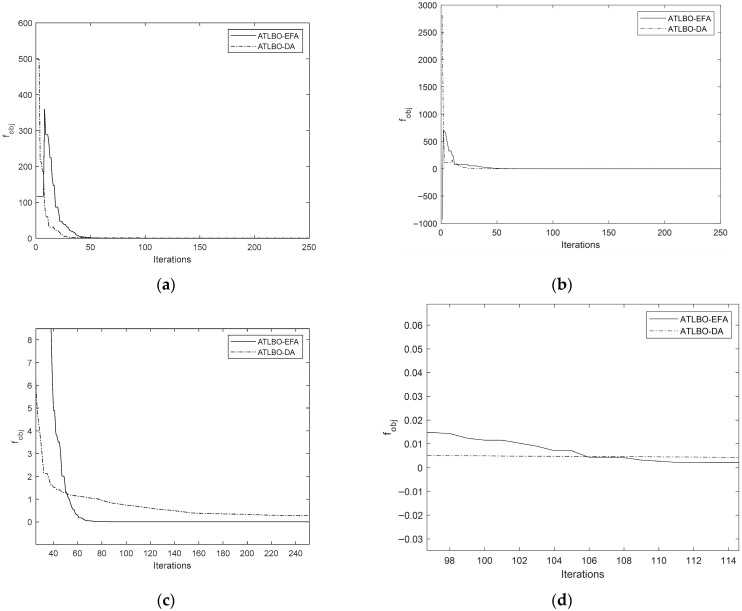
Search history of ATLBO-DA and ATLBO-EFA. (**a**) Problem 3. (**b**) Problem 4. (**c**) Enlarged search history of Problem 3. (**d**) Enlarged search history of Problem 4.

The convergence history plots comparing the original TLBO and the new ATLBO-EFA show that the search histories of ATLBO-EFA fluctuate more strongly than those of TLBO-DA, as shown in Figure 8 and Figure 9 (including the enlarges views). This extended fluctuation reflects the exploration capability of ATLBO-EFA.

Importantly, the incorporation of the Evenness Factor Archive (EFA) effectively prevents premature convergence, allowing the algorithm to maintain diversity and achieve superior optimization performance compared to the baseline approach.

**Figure 10:**
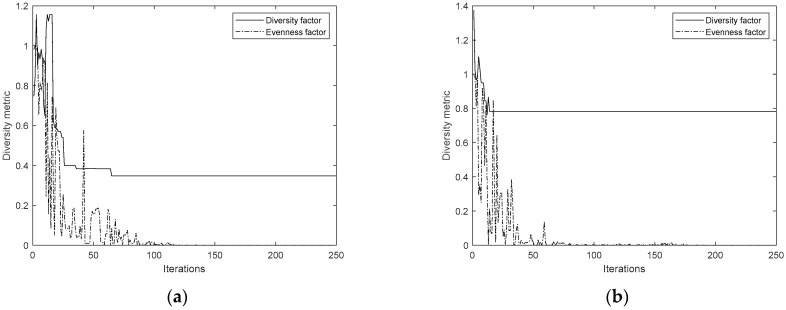

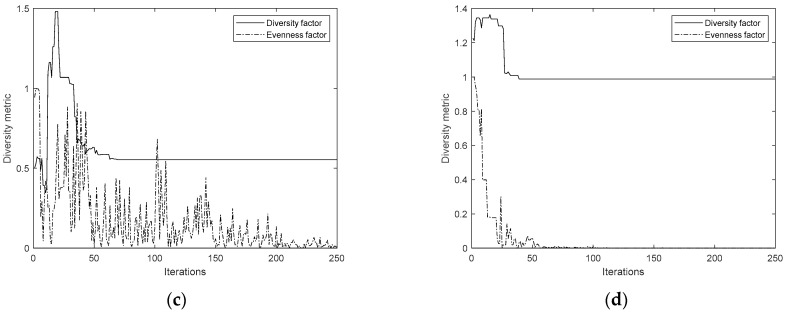
Diversity metric of ATLBO-DA and ATLBO-EFA plot over time. (**a**) Problem 1. (**b**) Problem 2. (**c**) Problem 3. (**d**) Problem 4.

A further look inside the diversity metric reveals that ATLBO-DA employs a diversity factor, while the ATLBO-EFA uses an evenness factor. Both metrics are used to control diversity of population, which is expected against premature convergence. As illustrated in Figure 10, convergence history of the best minimum values across the four problems highlight the role of these metrics in maintaining diversity and supporting effective exploration during optimization. The diversity metric is modified using 1/max(FD), where FD represents the diversity factor in Euclidean norm. This adjustment ensures the metric behaves directionally similar to the evenness factor (EV), with both tending toward zero—where lower values indicate better performance. The smaller 1/max(FD) means less diversity, while the evenness factor close to zero means greater evenness. Convergence history plots of all problems show the evenness factor fluctuates more strongly and for longer durations than the diversity factor. It means the diversity of population can be handled by evenness factors rather than by direct diversity factor. The sustained fluctuation of EV helps prevent premature convergence and supports the search for optimal solutions. In the ATLBO-DA case, fluctuations diminish after approximately 50 iterations, signaling reduced exploration. However, ATLBO-EFA maintains fluctuations until the end of the run, thereby sustaining diversity throughout the optimization process. This persistent variability explains why the new technique achieves superior results.

## 8. Conclusions

In conclusion, optimizing four-bar linkage path generation through a self-adaptive teaching–learning approach incorporating an evenness factor archive demonstrates significant potential. The ATLBO-EFA combined with an optimum path repairing technique significantly improves four-bar linkage path synthesis by minimizing path error and enhancing solution consistency. Across all cases, ATLBO-EFA achieved lower mean errors, best objective values, and reduced variability, consistently outperforming previous methods in aligning the actual and target paths. A non-parametric statistical analysis using the Friedman test confirms ATLBO-EFA to be the best-ranking method, with *p*-value (0.0455) < α (0.05). It can be summarized that ATLBO-EFA is the best performer for solving the four-bar path synthesis problems cases 1–4. This novel approach provides robust handling of complex constraints, making it both effective and efficient for systems requiring precise and reliable path generation.

For future work, this technique will be used to synthesize a variable camber mechanism to control a variable camber wing or morphing wing.

## Figures and Tables

**Table 1:** Comparative results of Problems 1 and 2.

Problem 1	With Optimum DL		Problem 2	With Optimum DL	
Parameters	ATLBO-DA	ATLBO-EFA	Parameters	ATLBO-DA	ATLBO-EFA
*r*_1_	41.3622	15.4197	*r*_1_	47.3319	47.3318
*r*_2_	8.4046	5.1258	*r*_2_	8.9594	8.9594
*r*_3_	24.3830	30.9560	*r*_3_	26.1415	26.1415
*r*_4_	28.3559	22.5307	*r*_4_	50.0000	50.0000
*r*_px_	3.7795	56.5099	*r*_px_	43.5296	43.5295
*r*_py_	43.5926	−60	*r*_py_	−27.9914	−27.9916
*x*_2_	54.1462	−57.2968	*x*_2_	16.8224	16.8224
*y*_2_	5.5719	40.5854	*y*_2_	−50.0000	−50.0000
*θ*_0_	0.3758	0.0013	*θ*_0_	0.8441	0.8441
*θ* ^1^_2_	0.3171	1.7766	**Mean**	9.888042	6.814966
*θ* ^2^_2_	0.5803	2.1189	**Min**	0.761388	0.761388
*θ* ^3^_2_	0.8350	2.4517	**Max**	16.90563	16.90563
*θ* ^4^_2_	1.1103	2.7785	**Std**	7.84176	7.325533
*θ* ^5^_2_	1.4531	3.1020	**Error**	0.3073	0.3073
*θ* ^6^_2_	2.3074	3.4283	*k*_op_	1.0609	1.2082
**Mean**	0.361816	0.092709			
**Min**	6.52 × 10^−5^	1.5 × 10^−6^			
**Max**	3.205011	1.03494			
**Std**	0.810662	0.259242			
**Error**	0.0026	4.58 × 10^−4^			
*k*_op_	1.7929	1.0449			

**Table 2:** Comparative results of the Problem case 3–4.

Case 3	With Optimum DL		Case 4	With Optimum DL	
Parameters	ATLBO-DA	ATLBO-EFA	Parameters	ATLBO-DA	ATLBO-EFA
*r*_1_	44.9664	79.1735	*r*_1_	3.9057	38.2658
*r*_2_	8.0475	8.0181	*r*_2_	0.2752	0.3898
*r*_3_	28.2160	50.5317	*r*_3_	7.4072	38.6554
*r*_4_	26.8741	42.6833	*r*_4_	3.8965	0.9075
*r*_px_	−6.3206	−10.6619	*r*_px_	1.5522	18.0488
*r*_py_	−0.7395	−3.1406	*r*_py_	1.3915	11.9765
*x*_2_	10.7622	10.9675	*x*_2_	1.6274	5.9630
*y*_2_	16.2596	21.0606	*y*_2_	−0.8826	−20.2262
*θ*_0_	0.7561	0.7024	*θ*_0_	1.2609	1.2325
*θ* ^1^_2_	6.5672 × 10^−4^	9.3801 × 10^−4^	*θ* ^1^_2_	1.2609	1.2325
*θ* ^2^_2_	0.7037	0.7023	**Mean**	0.558281	0.286989
*θ* ^3^_2_	1.4032	1.4053	**Min**	0.033655	0.029717
*θ* ^4^_2_	2.1189	2.1186	**Max**	2.541645	2.146459
*θ* ^5^_2_	2.8460	2.8310	**Std**	0.844748	0.403582
*θ* ^6^_2_	3.5603	3.5365	**Error**	0.039	0.037
*θ* ^7^_2_	4.2515	4.2280	*k*_op_	1.8124	1.1989
*θ* ^8^_2_	4.9292	4.9118			
*θ* ^9^_2_	5.6081	5.5956			
*θ* ^10^_2_	6.2809	6.2831			
**Mean**	1.165178	0.291322			
**Min**	0.00756	0.000954			
**Max**	17.94188	6.402288			
**Std**	3.768556	1.223671			
**Error**	0.0234	0.0086			
*k*_op_	1.0149	1.9105			

**Table 3:** Average ranking and *p*-value of performance index of ATLBO-DA and ATLBO-EFA achieved by Friedman test.

*p*-Value	Average Ranking of Both Techniques Friedman	
	ATLBO-DA	ATLBO-EFA
0.0455	2	1
	(2)	(1)

**Table 4:** Average running time(s) of ATLBO-DA and ATLBO-EFA applied to path synthesis problems.

Problems	ATLBO-DA	ATLBO-EFA
I	11.6897	27.8276
II	11.6207	27.3448
III	11.6207	27.5517
IV	11.7586	27.5862

## Data Availability

The data presented in this study are available in article.
